# Supplementary material for: Precocious centriole disengagement and centrosome fragmentation induced by mitotic delay
Source: Nat Commun. 2017 Jun 13;8:15803. doi: 10.1038/ncomms15803 (PMC5474744; doi:10.1038/ncomms15803)
Supplement: Supplementary Information — Supplementary Figures [file ncomms15803-s1.pdf]

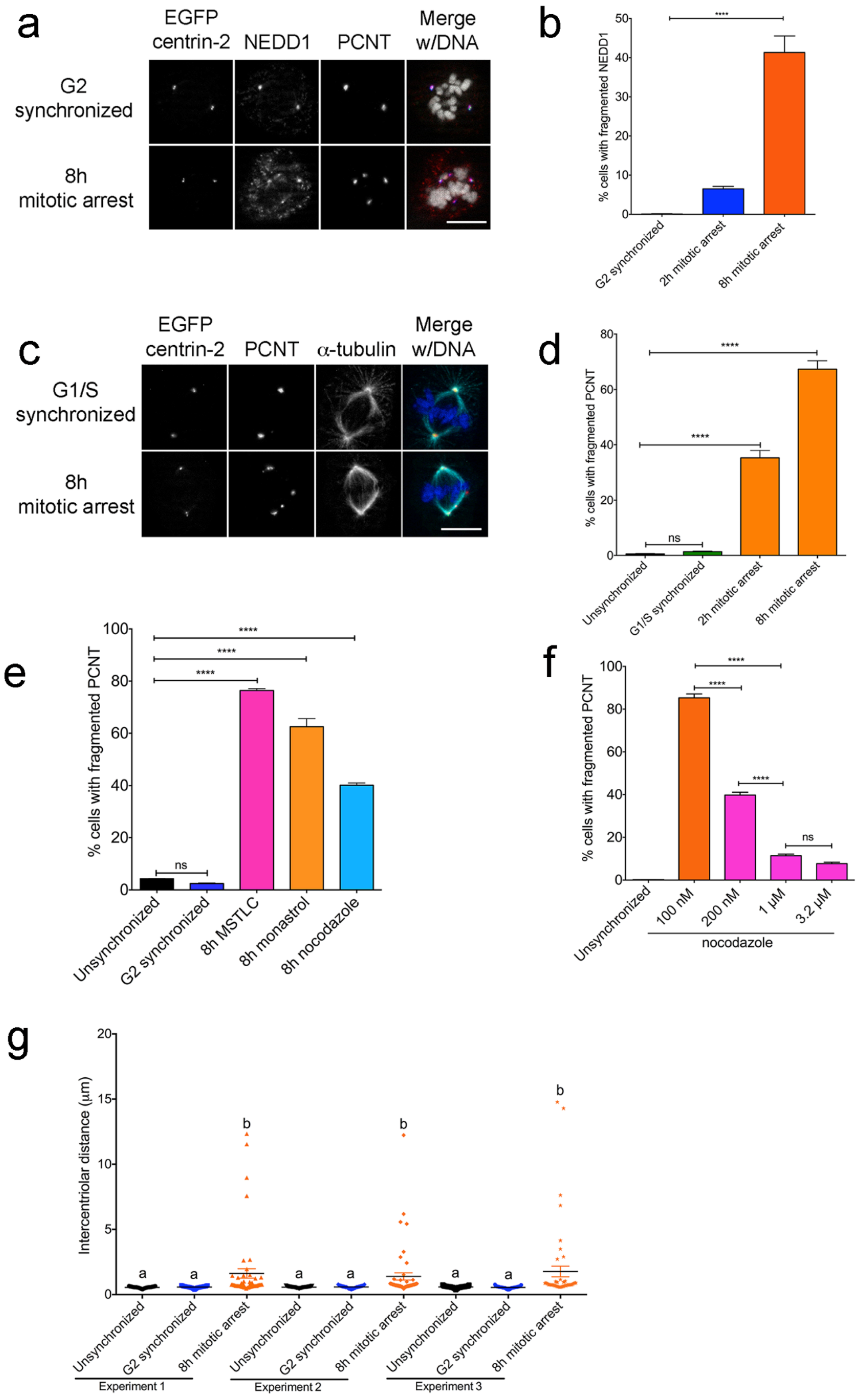
a
EGFP centrin-2
NEDD1
PCNT
Merge w/DNA
G2 synchronized
8h mitotic arrest
b
% cells with fragmented NEDD1
****
G2 synchronized
2h mitotic arrest
8h mitotic arrest
c
EGFP centrin-2
PCNT
α-tubulin
Merge w/DNA
G1/S synchronized
8h mitotic arrest
d
% cells with fragmented PCNT
ns
****
Unsynchronized
G1/S synchronized
2h mitotic arrest
8h mitotic arrest
e
% cells with fragmented PCNT
ns
****
Unsynchronized
G2 synchronized
8h MSTLC
8h monastrol
8h nocodazole
f
% cells with fragmented PCNT
****
ns
Unsynchronized
100 nM
200 nM
1 μM
3.2 μM
nocodazole
g
Intercentriolar distance (μm)
a
b
Unsynchronized
G2 synchronized
8h mitotic arrest
Experiment 1
Experiment 2
Experiment 3

Supplementary Figure 1. Related to Figure 1. Characterization of centrosome fragmentation in mitotically delayed RPE1 cells.

(a) Cells transiently transfected with EGFP centrin-2 (Green), synchronized in G2 and then either released to proceed into mitosis (Top row) or arrested for 8 hours with monastrol prior to release (bottom row). Cells were fixed and then probed for NEDD1 (Red), pericentrin (PCNT, blue) and DNA (white). Bar, 10 μm. (b) Quantification of NEDD1 fragmentation. (c and d) EGFP centrin-2 (Green)-expressing cells were synchronized using a double thymidine block, and then either released to proceed into mitosis (Top row) or arrested for 8 hours with monastrol prior to release (bottom row). Bar, 10 μm. PCM Fragmentation occurred in mitotically delayed cells in a manner comparable to cells synchronized in G2 (Figure 1c). (e) Quantification of centrosome fragmentation observed following prometaphase arrest with different spindle antagonists (5 μM MSTLC, 100 μM monastrol, 200 nM nocodazole) or (f) increasing concentrations of nocodazole. For panels b,d,e and f, error bars represent SEM for three replicate experiments per condition, n=300 cells, per experiment, and significance was determined by one-way ANOVA with Tukey-Kramer post-hoc test, **** $p \leq 0.0001$. (g) Measurements of intercentriolar distances from three experimental replicates, of which experiment 2 is depicted in Figure 1d. For the individual experiments, significant differences were calculated for each comparison using a non-parametric Kruskal-Wallis test ($p < 0.05$), and significant differences between samples were indicated with different lower case letters.

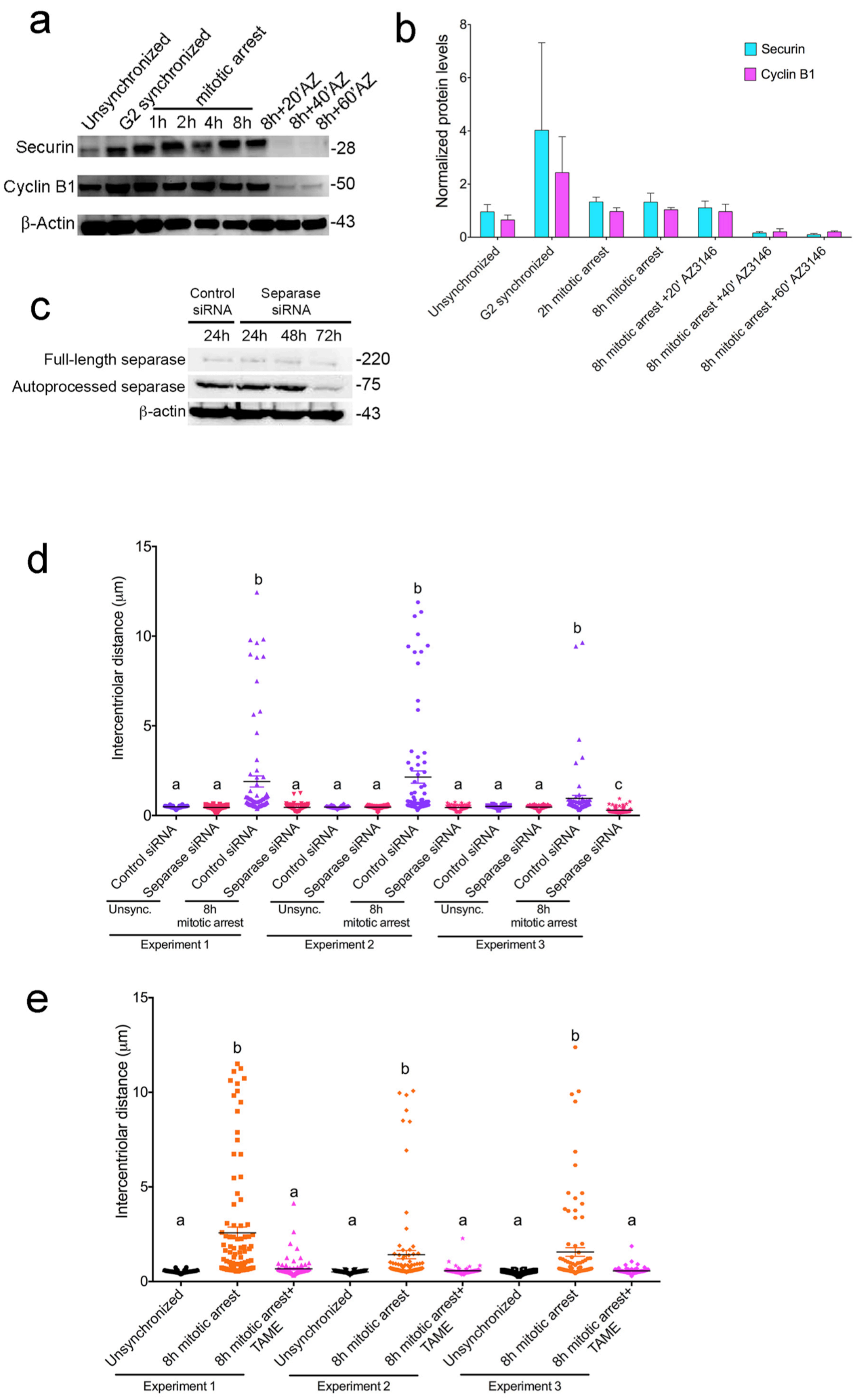

a
Unsynchronized
G2 synchronized
mitotic arrest
1h 2h 4h 8h
8h+20'AZ
8h+40'AZ
8h+60'AZ
Securin
Cyclin B1
β-Actin
-28
-50
-43
b
Normalized protein levels
Securin
Cyclin B1
Unsynchronized
G2 synchronized
2h mitotic arrest
8h mitotic arrest
8h mitotic arrest +20' AZ3146
8h mitotic arrest +40' AZ3146
8h mitotic arrest +60' AZ3146
c
Control siRNA
Separase siRNA
24h 24h 48h 72h
Full-length separase
Autoprocessed separase
β-actin
-220
-75
-43
d
Intercentriolar distance (μm)
Control siRNA
Separase siRNA
Unsync.
8h mitotic arrest
Experiment 1
Experiment 2
Experiment 3
e
Intercentriolar distance (μm)
Unsynchronized
8h mitotic arrest
8h mitotic arrest+ TAME
Experiment 1
Experiment 2
Experiment 3

Supplementary Figure 2. Related to Figure 3. Quantification of mitotic protein levels, separase depletion and intercentriolar distances.

(a) Whole cell securin or cyclin B levels were detected in cells that were either unsynchronized, arrested in G2, or synchronized in G2 and then released into monastrol for varying amounts of time. As a positive control for APC/C- mediated degradation, cells arrested for 8 hours with monastrol were treated with the Mps1 inhibitor AZ3146 (10 μM) for 20-60 minutes to allow for mitotic exit. (b) Quantification of the normalized protein levels, and error bars representing SEM for three replicate experiments. No significant differences were identified by one-way ANOVA with Tukey-Kramer post-hoc test. (c) RPE1 cells were transfected with control or separase siRNA for different time points and full-length and auto-processed forms of separase were detected by Western blotting. (d and e) Measurements of intercentriolar distances from three experimental replicates, of which experiment 2 is shown in Figure 3c and experiment 1 is shown in Figure 3f. For the individual experiments, significant differences were calculated for each comparison using a non-parametric Kruskal-Wallis test ($p < 0.05$), and significant differences between samples were indicated with different lower case letters.

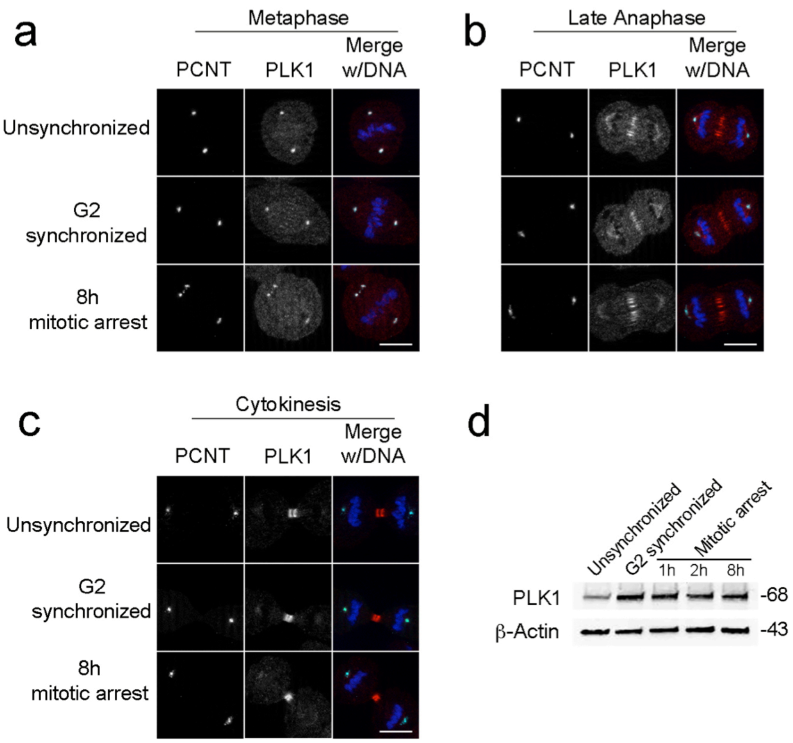

Supplementary Figure 3. Plk1 localization and stability during mitotic arrest. (a-c) G2-synchronized RPE1 cells were either allowed to directly progress into mitosis or were treated with monastrol for 8 hours before being released from prometaphase arrest to permit mitotic progression. Cells were fixed and then probed for PCNT (Cyan), PLK1 (Red) and DNA (blue). Bar, 10 μm. (D) Protein samples were harvested from unsynchronized, G2-synchronized, or mitotically-delayed cultures, and probed for PLK1 and actin.

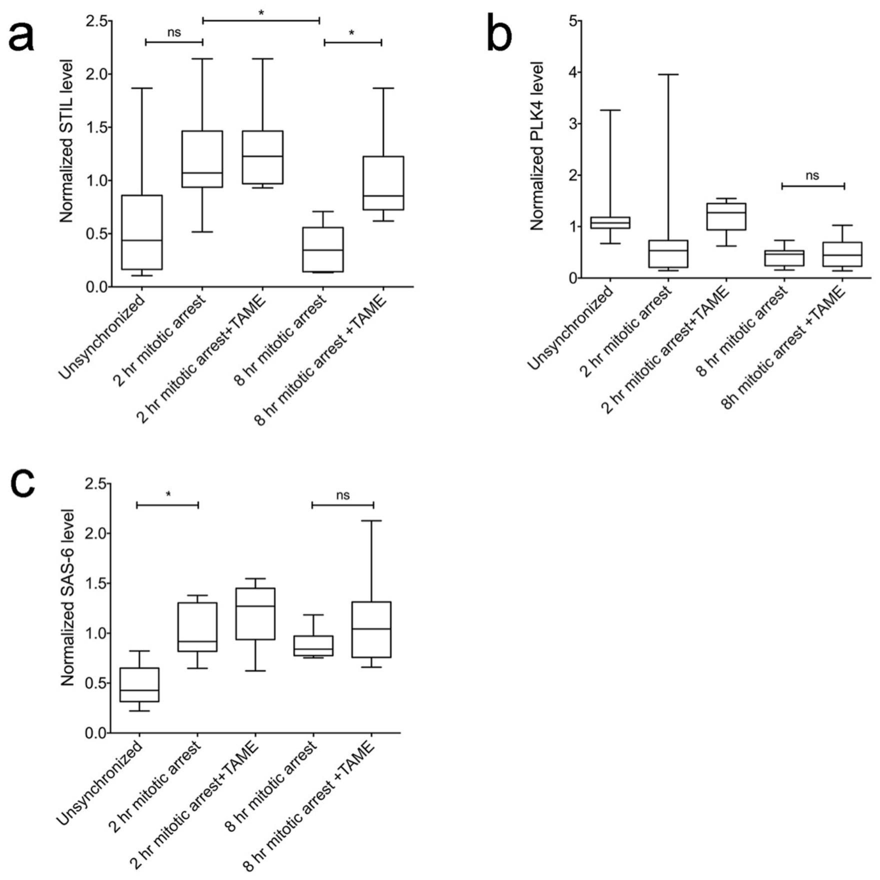

Supplementary Figure 4. Related to Figure 4. Quantification of procentriole markers in cells delayed in mitosis.

Quantification of normalized levels of STIL, PLK4 and SAS-6 for eight separate experiments as depicted in Figure 4. The upper and lower limits of the boxes demarcate the 25th and 75th quartiles, respectively, with the line denoting the median. Significance was determined by one-way ANOVA with Tukey-Kramer post-hoc test, * $p \leq 0.05$.

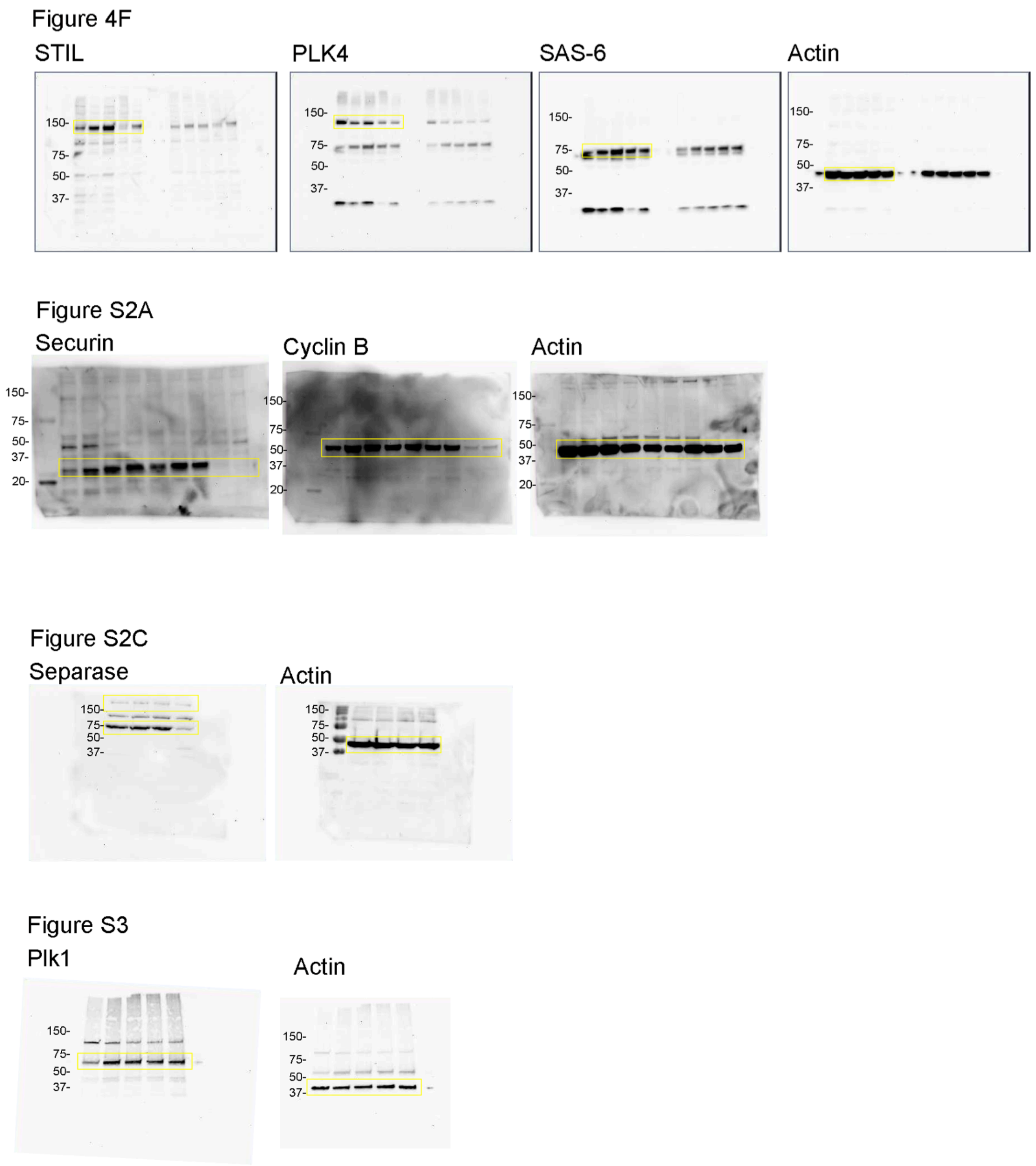

Supplementary Figure 5. Uncropped images of Western blots for Figures 4, Supplementary Figure 2 and Supplementary Figure 3. Yellow boxes denote areas cropped for figure generation.
